# Cascade dams altered taxonomic and functional composition of bacterioplankton community at the regional scale

**DOI:** 10.3389/fmicb.2023.1291464

**Published:** 2023-10-25

**Authors:** Xufei Jiang, Yan Liu, Rixiu Zhou, Tianyi Sun, Jingdan Cao, Shuqing An, Jiachen Shen, Xin Leng

**Affiliations:** ^1^School of Life Science and Institute of Wetland Ecology, Nanjing University, Nanjing, China; ^2^Key Laboratory for Information System of Mountainous Area and Protection of Ecological Environment of Guizhou Province, Guizhou Normal University, Guiyang, China

**Keywords:** cascade dams, regional scale, bacterioplankton, community assembly, environmental response, taxonomic diversity and functional groups

## Abstract

Dams are increasingly disrupting natural river systems, yet studies investigating their impact on microbial communities at regional scale are limited. Given the indispensable role of bacterioplankton in aquatic ecosystems, 16S rRNA gene sequencing was performed to explore how these communities respond to dam-influenced environmental changes at the regional scale in the Shaying River Basin. Our findings revealed that cascade dams create distinct environments, shaping bacterioplankton communities near the dams differently from those in natural rivers. In the upstream of the cascade dams, water quality was superior, while bacterioplankton community structure was simple with weak community interactions. In the midstream, nutrient and heavy metal content were increased, making bacterioplankton structures more susceptible to environmental changes. In the downstream of the cascade dams, water quality had a significant impact on the community and the bacterioplankton structures were highly complex. Additionally, environmental variables significantly influenced bacterioplankton functional groups. However, the response to these factors, as well as the interplay between functional and taxonomic diversity, varied markedly depending on the specific region of the cascade dams. We here delved into the effects of cascade dams on the taxonomic diversity and functional groups of bacterioplankton to provide a theoretical basis for segmentally regulating these dams.

## 1. Introduction

The construction of hydroelectric dams has led to severe and widespread disturbances in natural river systems (Zhao et al., 2012), creating distinct physical and chemical environments that disrupt nutrient transport and influence community structure and biodiversity (Wu et al., 2019; Maavara et al., 2020). Moreover, the proliferation of cascade damming has intensified pressures on rivers globally (Wang et al., 2018). In recent years, there has been a growing focus on the repercussions of dam construction on river biotic communities and ecological functions (Chen et al., 2018; Yuan et al., 2022). Nevertheless, considerable uncertainties continue to envelop the precise ramifications of cascade damming on the environment and river community structure within a watershed.

When delving into the mechanisms governing the assembly of biological communities in rivers subjected to dam regulation, it’s crucial to acknowledge that varying scales of investigation can yield distinct insights into the influences of environmental factors and dispersal constraints (Lange et al., 2018). Studies have indicated that on smaller, local scales, environmental factors tend to exert a more significant influence than dispersal limitations in shaping community structure (Yan et al., 2015; Li et al., 2023). Conversely, on larger scales, the constraints related to dispersal might surpass the impact of environmental variables on river organisms (Declerck et al., 2011; Lu et al., 2020; Wang et al., 2020b). Thus, when investigating ecosystem communities within cascade dams, it’s crucial to consider variations not only at the local scale but also at the regional scale.

Bacterioplankton, one of the most abundant forms of life on Earth, inhabit nearly every aquatic environment (Karner et al., 2001). Their diversity and dynamic behavior exert profound impacts on ecosystem material and energy cycles (Woese, 1994). Hydrological and aquatic environmental changes caused by cascade damming readily influence bacterioplankton as they are sensitive to their environment (Wang X. et al., 2020; Yang et al., 2020). This phenomenon has been extensively investigated in studies focusing on community taxonomy (Barberan et al., 2012; Banerjee et al., 2018). Research into functional diversity has also expanded, revealing that cascade dams not only alter microbial taxonomic composition but also influence ecosystem functions through their impact on functional traits (Yang, 2021). However, confining the examination of microbial responses to environmental changes solely through taxonomic composition or functional traits offers a limited perspective. At present, there remains a conspicuous lack of clarity concerning both taxonomy and functional interactions. Therefore, it is imperative to conduct a thorough and comprehensive investigation into the structural and functional interactions among bacterioplankton, as well as to scrutinize the discrepancies within watersheds. This approach is pivotal for the advancement of our comprehension of community structures and for the identification of effective strategies aimed at the restoration of ecosystems within regulated watersheds (Martini et al., 2021; Li et al., 2022).

For this study, 21 sampling sites were carefully selected from seven dams situated in the Shaying River Basin in China. Our analyses were structured to explore how bacterioplankton taxa and functional traits respond to environmental conditions and their interplay across different dam regions. Specifically, our analyses encompassed: (1) Assessing the environmental impact of cascade dams on a regional scale. (2) Analyzing the diverse variations in the composition and assembly mechanisms of bacterioplankton community structures at a regional scale. (3) Investigating the influence of environmental factors on the functional groups of bacterioplankton and exploring potential variations in the relationship between taxonomic and functional groups. This comprehensive analysis aims to underscore the importance of sub-regional management in the context of communities surrounding cascade dams.

## 2. Methods

### 2.1. Study site and sampling

**Figure 1:**
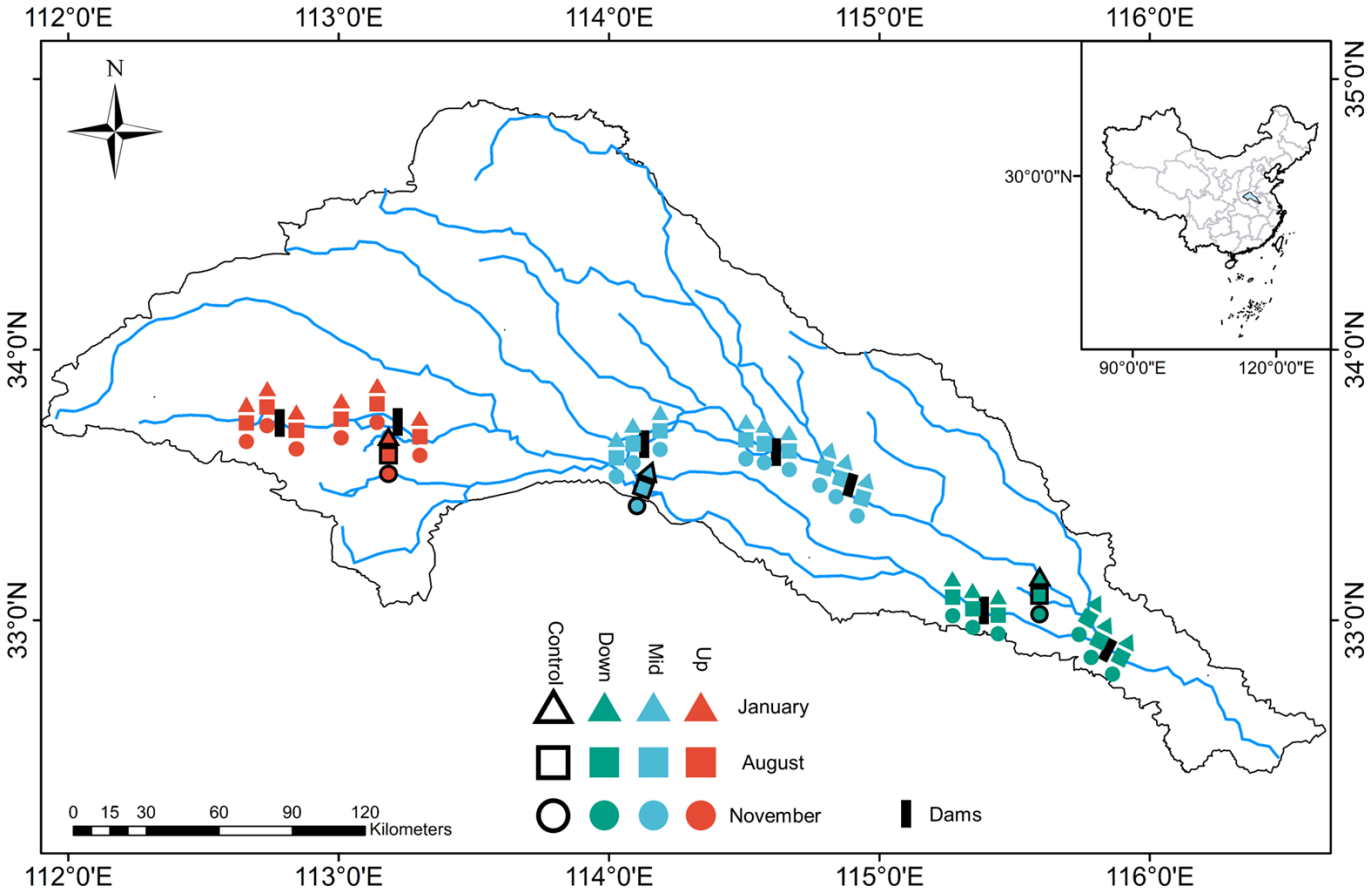
Sampling sites in upstream (Up), midstream (Mid), downstream (Down), and control group in the Shaying River Basin.

Sampling surveys were conducted in the Shaying River Basin (Figure 1), which is located in central Henan Province and northwest Anhui Province, China (111°56′44″ − 116°31′07″ E, 32°29′24″ − 34°57′15″ N). The river is 619-km long and has a watershed area of 36,651 km^2^, accounting for approximately 88% of the total area of the Huaihe River Basin. This basin contains 72 dams, including 16 large-sized and 56 small-or medium-sized dams, and hence is an ideal watershed for studying the effects of dams on the community structure of the river ecosystem (Zhang and Zhang, 2014; Shen et al., 2023). In this study, seven dams in the basin studied was selected for sample collection for three times in 2022, including two upstream reservoirs (Up), three midstream floodgates or ship locks (Mid), and two downstream floodgates (Down) at the regional scale. Three sample sites were selected for each dam, including upstream of the dam (>5 km away from dam and within the thalweg of the flowing stream), center of the reservoir, and downstream of the dam (>5 km leaving the plunge pool) composed as the local scale. In this way we aim to explore the impacts of cascade dams on communities at different spatial scales (Yongyong et al., 2007; Zhang et al., 2011). Furthermore, to determine whether the dams affected the community structure, a tributary not regulated by the dams was selected as a control group at different locations in the three watershed locations.

For each sampling time, freshwater water samples were collected using 10-L Niskin bottles from the surface layer at 0.5 m depth at each sampling site. The samples were collected in three replicates and passed through microporous membranes (pore size: 0.22 μm; diameter: 50 mm; Millipore, USA) for molecular analyses. Microorganisms attached to the membranes were then microbiologically analyzed. The microbial filter membrane samples were stored at −80°C until DNA extraction.

### 2.2. Water quality and heavy metal indicators

River environmental indicators were measured through field collection and laboratory measurement. Water temperature (WT), dissolved oxygen concentration (DO), pH, electrical conductivity (EC), oxidation reduction potential (ORP), total dissolved solids (TDS) and turbidity (NTU) were measured using portable meters HACH HQ40D and HACH 2100q.

Different sets of water samples were collected and analyzed in the laboratory within 24 h of sampling. Chemical oxygen demand (COD), ammonia nitrogen (NH_4_^+^-N), phosphate (RP), total nitrogen (TN), and total phosphorus (TP) were measured using a HACH digester (HACH-DRB200) and spectrophotometer (HACH-DR3900). At each sampling site, we collected 1-L of water samples, which were subsequently utilized to determine the heavy metal content. This analysis included the measurement of aluminum (Al), iron (Fe), lead (Pb), mercury (Hg), cadmium (Cd), chromium (Cr), arsenic (As), and zinc (Zn). We conducted these analyses using an Inductively Coupled Plasma Mass Spectrometer (ICP-MS) known as ICAP-Q, manufactured by Thermo Fisher Scientific.^1^ In this process, one method blank was included for every five samples, and data quality was monitored using standard samples (GNM-M2610144–2013, China). For the subsequent analysis, normalization was performed for removing the magnitude difference between different factors. One-way analysis of variance (ANOVA) was used to explore whether the environments differed between different regions. The results were visualized in ArcGIS (ESRI, Redlands, CA, USA) by using Kringing interpolation.

### 2.3. DNA extraction and 16S rRNA amplicon sequencing

DNA was extracted from 72 freshwater samples by using the FastDNA SPIN Kit (MP Biomedicals, USA) following the manufacturer’s instructions. Shanghai Majorbio Technology (Shanghai, China) performed sequencing on the Illumina MiSeq platform using paired-end sequencing. The primers 338F “ACTCCTACGGGAGGCAGCAG” and 806R “GGACTACHVGGGTWTCTAAT” were employed to PCR amplify the 16S rRNA gene covering the V3–V4 region (Shi et al., 2020). We performed sequencing for the purified amplicons using UPARSE (Edgar, 2013), high-quality sequences were clustered into operational taxonomic units (OTUs) at the 97% sequence similarity level. All taxonomy tables were filtered to exclude OTUs classified as chloroplasts and mitochondria. Finally, the average sequence length we obtained was 418 bp and the sequence data were normalized to 48,134 sequences per sample. Sequencing data of this study has been submitted to the NCBI database with the BioProject ID PRJNA1005440.

### 2.4. Data analysis

#### 2.4.1. Community structure analysis

The bacterioplankton community composition across various sampling sites was quantified, and both abundance and Shannon diversity were calculated using the online Majorbio Cloud Platform.^2^ To assess differences at local and regional scales, one-way analysis of variance (ANOVA) was conducted. Non-metric multidimensional scaling (NMDS) analysis, based on Bray–Curtis dissimilarity, was employed to visualize beta diversity. Additionally, Permutational Multivariate Analysis of Variance (PERMANOVA) (Anderson, 2001) was applied to explore variations in beta diversity between control and dam groups at different watershed scales. These analyses were carried out using R software version 4.2.3.

Co-occurrence network analysis was performed using Networkx to decipher community interactions among the upstream, midstream, and downstream dams and detect keystone species (Gotelli and McCabe, 2002; Deng et al., 2012). We established co-occurrence networks of microbial communities across the three regions using the ‘igraph’ package (Csárdi and Nepusz, 2006). Subsequent modular analysis, evaluation of topological properties, and network visualization were performed using Gephi (version 0.10.1). Keystone taxa were identified with high degrees (>100) and low betweenness centrality values (<3,000), as these characteristics often indicate key players within the community (Ma et al., 2016; Lu et al., 2022).

#### 2.4.2. Matrix regressions and redundancy analysis

To explore the potential impact of water quality, heavy metal indicators, and geographical distance on bacterioplankton communities within dams, we employed univariate matrix regression analysis. For this purpose, we utilized Bray–Curtis dissimilarity on log-transformed bacterial abundance data. Water quality disparity, heavy metal disparity, and geographical distance were calculated using Euclidean distance metrics. The analysis was carried out utilizing the ‘vegan’ package (Oksanen et al., 2020) and the ‘adespatial’ package (Dray et al., 2021) within the R environment. Concurrently, we employed redundancy analysis (RDA) using the ‘vegan’ package in R to uncover influential factors that shape community structure, as well as to pinpoint species particularly responsive to environmental variables. It’s noteworthy that the control group was excluded from the aforementioned analyses.

#### 2.4.3. Bacterial functional gene groups

Dams impede the flow of essential nutrients, including carbon, phosphorus, nitrogen and silicon, along river networks (Maavara et al., 2020). To investigate the impacts on bacterioplankton, we annotated OTUs with functional information by cross-referencing them with the FAPROTAX database (Louca et al., 2016). The majority of annotated species could be assigned to groups actively involved in essential ecological functions, such as the biogeochemical cycling of carbon, nitrogen, and sulfur, as well as the degradation of organic matter (Merloti et al., 2019) in different regions of the cascade dam-regulated system. Correlation analysis was then employed to investigate the relationship between these functional groups and the aquatic environment. Only functional groups associated with organic matter and carbon transformation, nitrogen transformation, and sulfur transformation were retained for further analysis. We quantified the abundances and occupancies of these functional groups to unveil regional-scale differences in cascade dams. Spearman correlation analyses were conducted to explore the connections between environmental variables and functional groups. These analyses were conducted using the ‘Hmisc’ and ‘psych’ packages in R.

#### 2.4.4. Structural equation modeling

To examine direct and indirect relationships among all variables in the model, we employed structural equation modeling (SEM, Gao et al., 2021). For our study, we aggregated environmental variables significantly correlated with community differences in RDA, normalizing their data to derive water quality (WQ) and heavy metal (HM) indices. Additionally, we employed principal component analysis (PCA) to downscale alpha diversity and functional gene groups related to nitrogen transformation, sulfur transformation, organic matter and carbon transformation. The first principal component from each PCA, explaining over 60% of variance, was selected (Guo et al., 2013; Gao et al., 2021). NMDS1 represented beta diversity. SEM models were constructed for upstream, midstream, and downstream dams, as well as for the control group, utilizing the ‘lavaan’ package in R. Different conceptual models were evaluated, ultimately selecting the one with a comparative fit index (CFI) greater than 0.95 and a root mean square error of approximation (RMSEA) lower than 0.05.

## 3. Results

### 3.1. Environmental characteristics

**Figure 2:**
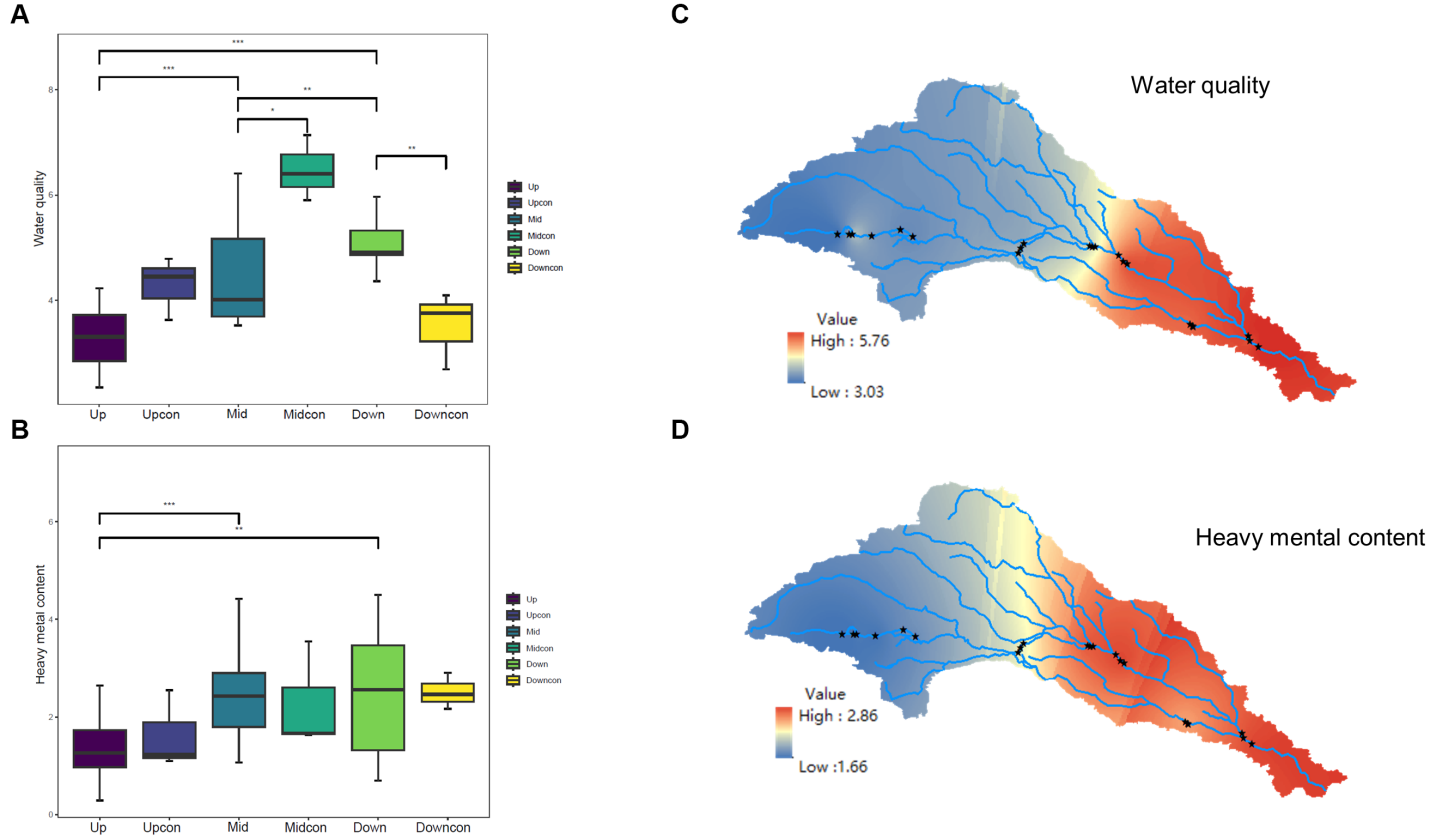
Environmental disparities between the dam-regulated rivers and the control group in the Shaying River Basin. **(A)** Comparison of water quality between the dam-regulated sampling points and the control group, revealing significant intergroup differences. **(B)** Comparison of heavy metal content between the dam-regulated sampling points and the control group. **(C)** Kringing interpolation depicting water quality across the entire watershed. **(D)** Visual representation of the variations in heavy metal content across the entire watershed. *means *p* < 0.05, **means *p* < 0.01, ***means *p* < 0.001.

One-way ANOVA and post-hoc tests revealed that EC and TDS were significantly different at the regional scale of cascade dams. Additionally, TN, AS, and Cr varied between the upstream location and the other two stream locations. NTU, TP, and NH_4_^+^-N differed statistically between the upstream and downstream dams. Cd was only different between the upstream and midstream dams (*p* < 0.05, Supplementary Table S1). A summation of the normalized environmental variables gave us water quality and heavy metal content. No disparity in water quality was observed between the upstream dam and the upstream control group, whereas water quality at both the midstream and downstream regions differed significantly from that in the respective control groups (*p* < 0.05, Figures 2A,B). The interpolation plots constructed by ArcGIS also showed a significant accumulation near the dams of water quality and heavy metals from upstream to downstream in the cascade dam-controlled rivers (Figures 2C,D).

### 3.2. Effects of dams on the community structure of bacterioplankton

**Figure 3:**
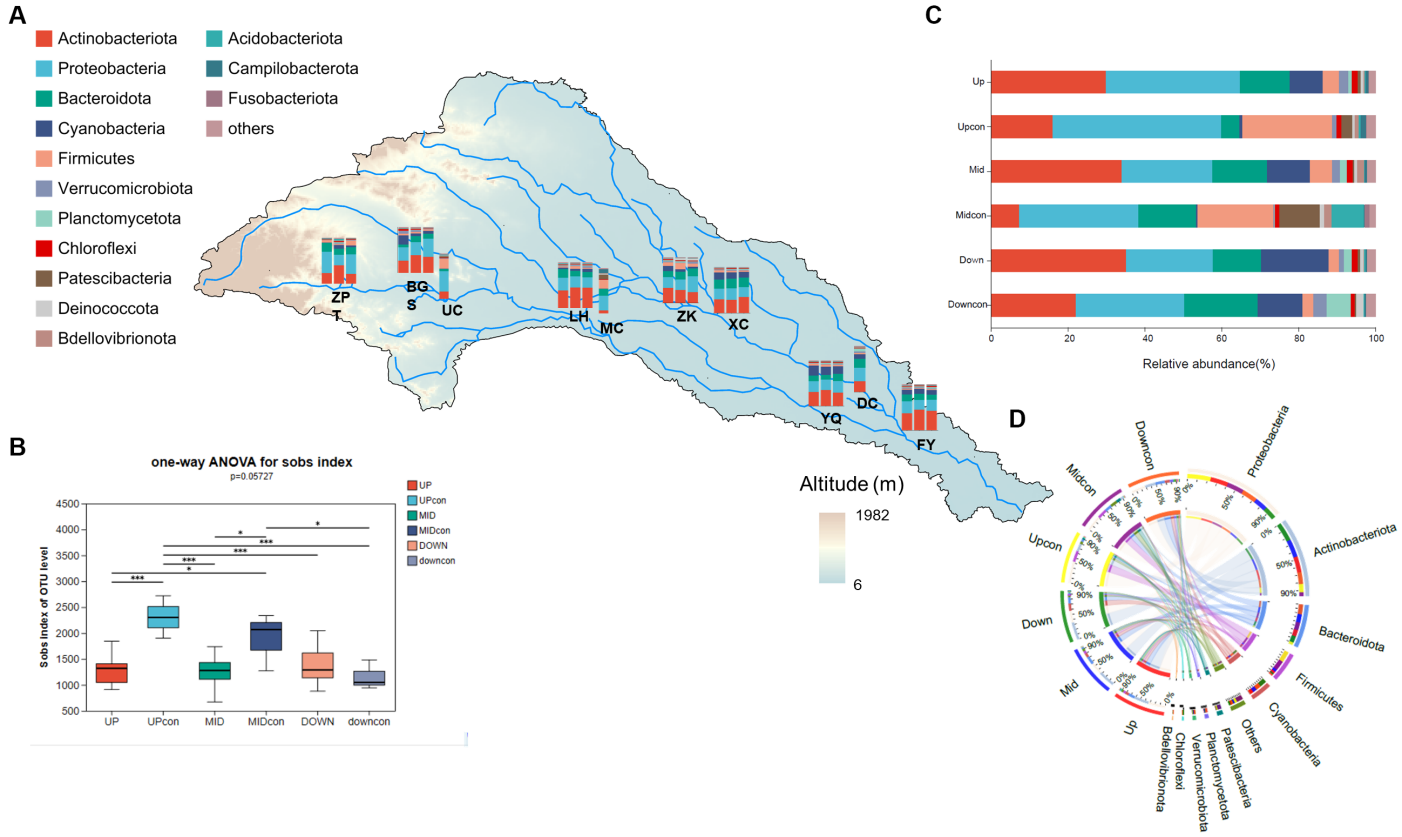
Bacterioplankton community structure in the cascade dam-regulated basin. **(A)** Taxonomic groups at the phylum level across the seven dams and three control groups. **(B)** Variations in the observed abundance among the different groups. **(C)** Comparison of taxonomic groups at the phylum level at the regional scale of the watershed locations and the control groups. **(D)** Circos plot illustrating the proportional distribution of dominant species in the regional scale and control group samples as well as the distribution proportions of these dominant phylum across the different groups. *means *p* < 0.05, **means *p* < 0.01, ***means *p* < 0.001.

In total, 10,220 OTUs were obtained for the bacterioplankton, including 65 phyla, 198 classes, 486 orders, and 1,824 genera. The abundant bacterial phyla were Actinobacteria (30.6%), Proteobacteria (27.7%), Cyanobacteria (11.1%), Bacteroidota (10%), Firmicutes (6%), and Verrucomicrobiota (1.9%). These phyla contributed more than 90% to all the communities. Our results revealed no significant differences in both the abundance and α-diversity of bacterioplankton among the local scales (Figure 3A; Supplementary Figure S1, *p* = 0.48), whereas the community structure varied significantly between the regional scales (Figures 3B,D), especially in the upstream reservoir area and the middle reaches serving as a storage dam and a run-off-river dam, respectively. Proteobacteria was the dominant phylum in the upstream control group (43.84%) and in the upstream reservoir area (34.81%) (Figure 3D). At the same time, the abundance and Shannon diversity index in the upstream control group were both significantly higher than those in the upstream reservoir area (Figure 3C). In the midstream, Actinobacteria was the dominant phylum (34.06%), but both the abundance of bacterioplankton and α-diversity were lower than those in the midstream control group (Figure 3B). Meanwhile, no significant differences were noted in abundance and α-diversity between the downstream dam and downstream control group.

**Figure 4:**
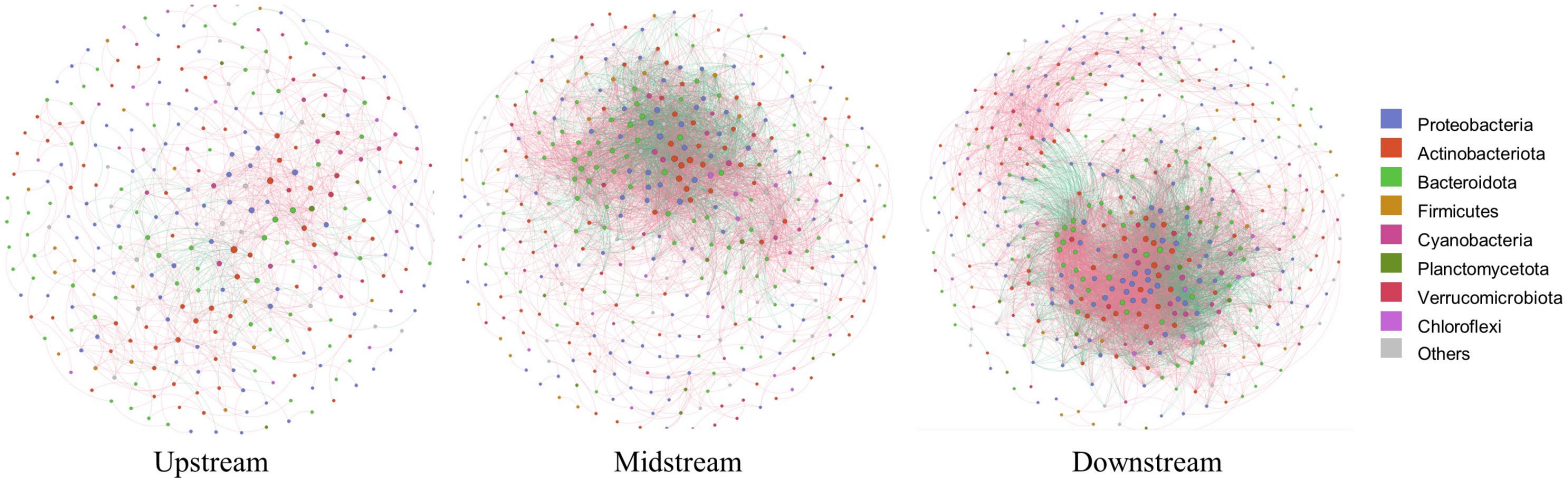
Co-occurrence networks of bacterioplanktons in the upstream, midstream, and downstream regions of the watershed, where each node represents an OTU; color-coded according to their taxonomic classifications. Node sizes reflect OTU abundances, while edge thicknesses reflect the strengths of correlations among the OTUs. Red and green edges signify positive and negative correlations between OTUs, respectively.

Within the watershed regulated by cascade dams, we evaluated the β-diversity of the community through PERMANOVA analysis. The permutation test indicated that the most significant differences were observed at the regional scale (Table 1, *p* = 0.006). Notably, the co-occurrence network also highlighted noteworthy variations at this same regional scale. Specifically, the upstream co-occurrence network comprised 325 nodes connected by 932 edges, albeit without any keystone species identified. The midstream co-occurrence network, on the other hand, featured 387 nodes and 4,801 edges, with 5 keystone species identified. Remarkably, 4 of these keystone species belonged to the Actinobacteriota family, with the notably abundant keystone species being *Ilumatobacteraceae* and *Sporichthyaceae* (Supplementary Table S2). Moving downstream, the co-occurrence network exhibited 427 nodes connected by 8,151 edges, boasting 48 keystone species. Impressively, the majority of these keystone species, accounting for 43.75%, belonged to the Actinobacteriota. Positive relationships among species were depicted with red-colored links, while negative relationships were denoted with green-colored links (Figure 4). In the upstream region, positive correlations constituted a significant 86.48% of all links. In the midstream, this proportion decreased to 65.32%. Interestingly, the downstream network displayed a marked presence of negative correlations, reaching 38%. This observation suggests heightened competition or predation among distinct taxonomic groups within this particular region.

**Table 1:** PERMANOVA analysis revealing differences in the bacterioplankton community structure between different scales.

Scales	Sums of Sqs	Mean Sqs	*F*-model	*R* ^2^	*P*-value	*P*-adjust
Regional scale	2.46	0.49	1.85	0.12	0.002	0.006
Dam scale	3.52	0.39	1.47	0.18	0.009	0.0135
Local scale	5.25	0.23	0.74	0.26	0.993	0.993

### 3.3. Bacterioplankton community assembly mechanisms under cascade dam influence

**Figure 5:**
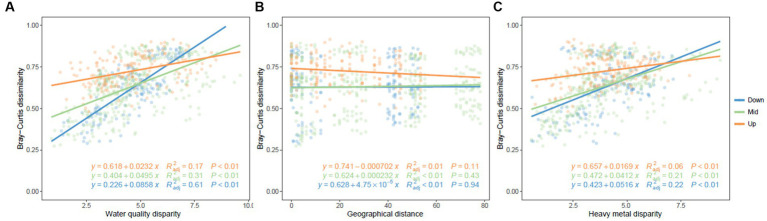
Pairwise distance matrices of community dissimilarity associated with water quality, geographical distance, and heavy metal disparity. Red, green, and blue represent **(A)** upstream, **(B)** midstream, and **(C)** downstream regions, respectively.

No significant relationship was observed between community dissimilarity and geographical distance (Figure 5). Notably, as variations in water quality and heavy metal content escalated, the heterogeneity of bacterioplankton communities likewise displayed a substantial increase. It’s interesting to note that in the downstream region, the community structure exhibited a markedly stronger correlation with water quality and heavy metal content, as compared to the midstream and upstream regions (Figure 5).

**Figure 6:**
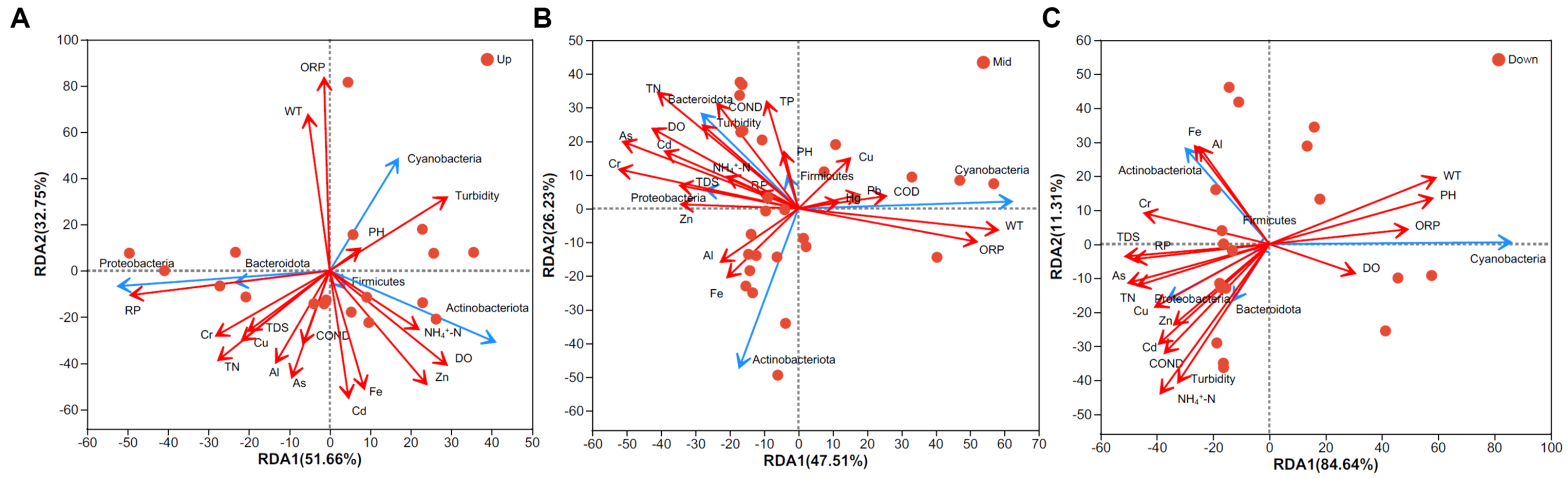
Redundancy analysis. **(A)** Upstream watershed, **(B)** midstream watershed, and **(C)** downstream watershed. Red points denote sampling sites, red arrows represent environmental variables, and blue arrows indicate taxonomic groups at the phylum level.

After conducting the VIF analysis, 17 variables were selected for RDA. WT and ORP were found to be crucial environmental variables influencing the bacterioplankton community composition in the upstream (Figure 6A; Supplementary Table S3). Additionally, Cyanobacteria was strongly and positively correlated with both WT and ORP. RP significantly influenced Proteobacteria. In the midstream region, WT and ORP were significantly and positively correlated with Cyanobacteria, while TN, As, Cr, and DO significantly impacted Bacteroidota and Proteobacteria (*p* < 0.05, Figure 6B). In the downstream region, a greater number of environmental variables significantly influenced the bacterioplankton community structure. For instance, pH, WT, and ORP exhibited significant effects on Cyanobacteria, while Fe, Al, and Cr were positively correlated with Actinobacteriota. NH_4_^+^-N, TDS, turbidity, COND, and heavy metals were significantly correlated with the community structure in the downstream region (Figure 6C; Supplementary Table S3).

### 3.4. APROTAX bacterial functional groups and environmental response

The FAPROTAX analysis revealed discernible distinctions in metabolic potential pertaining to carbon (C), nitrogen (N), and sulfur (S) cycling across the three regions of the cascade dam-regulated system. Within the upstream region, processes associated with sulfur and nitrogen transformation exhibited significant elevation in comparison to the middle and downstream areas. In the midstream of the cascade dam regions, the highest functional bacterial community abundance was linked to aerobic chemoheterotrophy and chemoheterotrophy. Moving downstream, the functional groups tied to carbon transformation displayed heightened abundance, particularly functions like photoautotrophy and oxygenic photoautotrophy.

**Figure 7:**
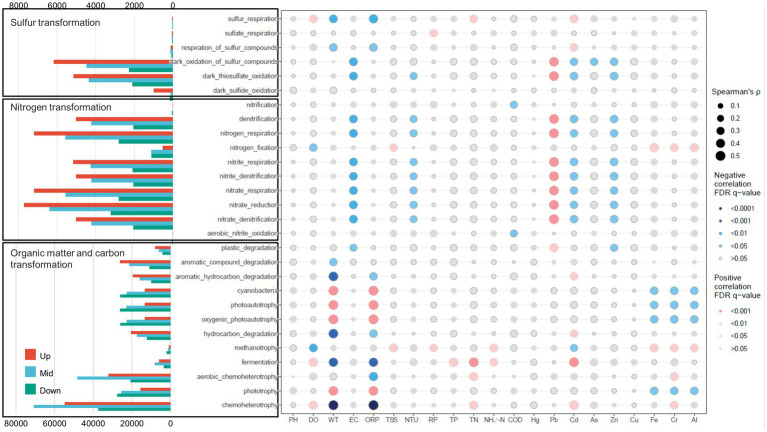
Distribution of bacterioplankton functional groups in upstream, midstream, and downstream dam-regulated watersheds, along with the correlation of each functional trait with environmental variables. Blue and red points indicate negative and positive correlations, respectively. The size of the points represents the strength of the correlation.

The influence of various environmental factors in shaping the functional microbial groups within dam-regulated rivers became apparent. The outcomes indicated that a majority of functional groups related to organic matter and carbon transformation exhibited significant correlations with variables like water temperature (WT) and oxidation–reduction potential (ORP) (*p* < 0.001, Figure 7). Moreover, these functional groups displayed correlations with Cd, Fe, Cr, and Al. Concerning nitrogen transformation, functional groups were found to be notably inversely correlated with electrical conductivity (EC), turbidity (NTU), Cd, and Zn, yet positively correlated with Pb. For sulfur transformation, dark thiosulfate oxidation and dark oxidation of sulfur compounds demonstrated negative correlations with EC, NTU, Cd, and Zn, but a positive correlation with Pb. Similarly, sulfur respiration and respiration of sulfur compounds exhibited negative correlations with WT and ORP, while showing a positive correlation with Cd (Figure 7).

### 3.5. Correlation between environmental variables, taxonomic diversity and functional groups

In the upstream region of cascade dams, a robust set of correlations was observed between community taxonomic diversity and functional structure. Specifically, water quality and various transformation processes, such as carbon and sulfur transformation, exhibited positive correlations with β-diversity. Conversely, nitrogen transformation and α-diversity displayed negative correlations in this context. Moving to the midstream region, characterized by high urban density and pronounced eutrophication, heavy metal content exhibited a significant negative correlation with community β-diversity. Furthermore, water quality was negatively correlated with sulfur transformation, carbon transformation, and α-diversity.

**Figure 8:**
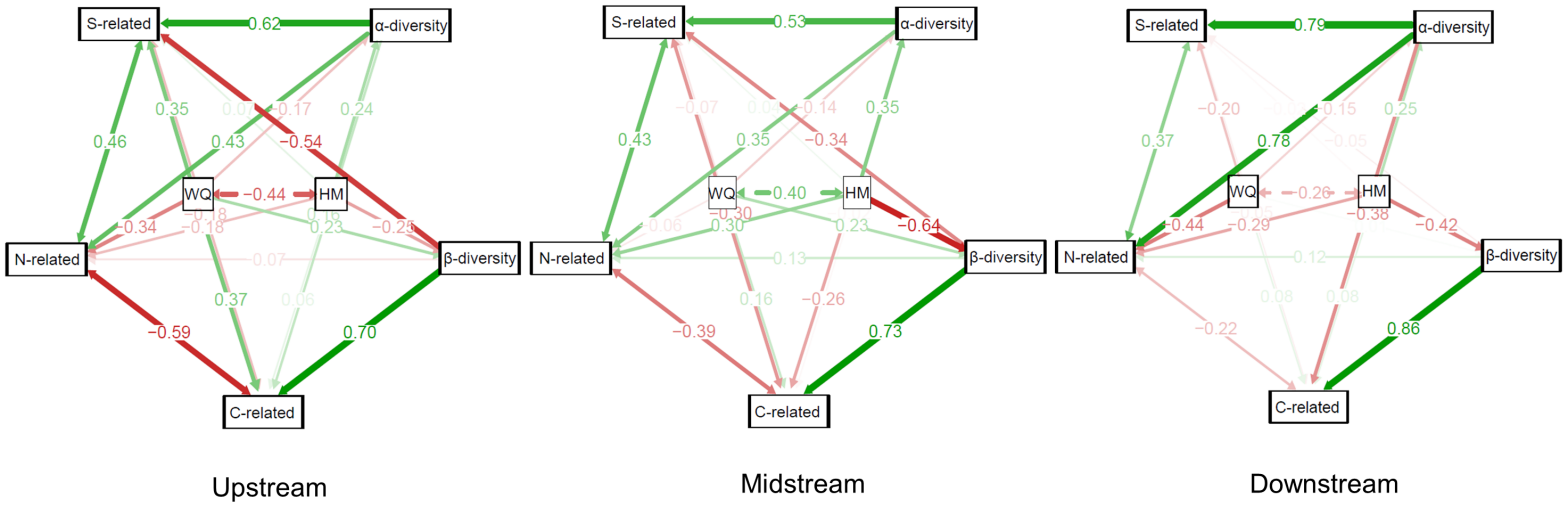
Structural equation models illustrating the direct or indirect impact of the environmental variables water quality and heavy metal content on the taxonomic and functional diversities of bacterioplankton, as well as the interrelations between the taxonomic and functional groups. Red and green lines indicate negative and positive relationships, respectively. The darkness of the line color indicates the strength of the correlation.

In the downstream region, an interesting pattern emerged where taxonomic diversity demonstrated a significant positive correlation with functional groups. Within this context, water quality and heavy metal content predominantly exerted negative impacts on the bacterioplankton community. Notably, heavy metal content exhibited significant negative correlations with both community taxonomic diversity and bacterial carbon transformation. Additionally, water quality displayed significant negative correlations with bacterial content associated with nitrogen transformation (Figure 8).

## 4. Discussion

### 4.1. Effects of cascade dams on water environmental variables

The impact of water environmental variables due to cascade dams was emphasized in our study. Notably, downstream accumulation of organic matter was exacerbated by these dams. The cumulative effects of cascade dams were intricate, not adhering to a simple addition or subtraction principle (Shi and Qin, 2023). Our findings illustrated a significant deterioration in the water quality of downstream areas of rivers regulated by cascade dams. Previous research has indicated that dams could mitigate downstream eutrophication pressure by retaining nutrients (Yamamoto, 2003), a phenomenon notably observed in large reservoirs located upstream. Nevertheless, on the watershed scale, particularly in relation to floating organic matter and minute suspended particles, there was a higher propensity for accumulation downstream through cascade dam spillways, contributing to heightened pollution levels (Tang et al., 2023).

### 4.2. Effects of damming on taxonomy and diversity of bacterioplankton

Our investigation underscored that, when compared to rivers unaffected by dams, the bacterioplankton community structure near dams demonstrates reduced diversity and increased uniformity, particularly upstream. This finding is consistent with previous research suggesting that dams can induce alterations in bacterioplankton composition and distribution by modifying flow patterns and nutrient availability (Li et al., 2019). Within the scope of our study, this trend was notably pronounced in two extensive upstream reservoirs, where prolonged water retention and increased storage depth could have amplified the hydraulic load of reservoirs, ultimately reducing the diversity of bacterioplankton (Lindström et al., 2005; Meiling et al., 2020). Moreover, beyond the localized impacts of individual dams, our comprehensive analysis underscored the intricate effects of cascading dams on the bacterioplankton community structure at the regional scale (Nogueira et al., 2010). Notably, this influence was most conspicuous in the notably augmented variability of β-diversity. In light of these complexities, a broader regional perspective, as opposed to a confined local scale, becomes essential when studying river ecosystems under the regulation of cascade dams (Zhang et al., 2010; Gilmar et al., 2011). Co-occurrence network analysis further illuminated that species interactions among bacterioplankton exhibited marked differences at the regional scale. Of particular interest, the prevalence of keystone species surged progressively from the upstream to the downstream segments of the cascade dams. In the upstream, the bacterioplankton network displayed a delicately simplistic configuration. Our conjecture is that the prolonged water retention within upstream reservoirs could have contributed to a debilitated stability within the bacterioplankton community (Wang et al., 2021). Conversely, downstream interactions within the community demonstrate greater complexity. The network revealed a surge in keystone species count and an elevation in negative edges proportion, signifying heightened competition and the potential reinforcement of network stability under disturbances (Berry and Widder, 2014; Coyte et al., 2015).

### 4.3. Influence of environmental variables on bacterial communities in cascade dammed rivers

Our study elucidated that, in the context of cascade dammed rivers, environmental variables held a predominant sway over the assembly mechanisms of bacterioplankton at the regional scale, surpassing the influences of diffusion limitations. This corroborates the findings of earlier investigations that underscore the significance of environmental variables as crucial drivers of planktonic species turnover (Amend et al., 2016; Xiao et al., 2021). For instance, the interplay of water temperature variations (Mai et al., 2022) and the accrual of heavy metals and nutrients, a consequence of dam reservoirs, emerged as pivotal in shaping the community assembly (Zhao et al., 2022). In the upstream region, water temperature stood out as a principal factor. Notably, extensive reservoirs, characterized by prolonged water retention periods, exacerbated temperature stratification (Weike et al., 2006). Further amplifying its impact was the discharge of colder water during dam events, imparting a transformative influence on the bacterioplankton community structure *via* temperature fluctuations (Zhang et al., 2014). Notably, ORP emerged as another influential factor, with strong correlations tying it to COD removal and nitrification. This link suggested a potential impact on bacterial community structure and functional traits, albeit to a limited extent (Su and Peng, 2011). In the midstream, TN, AS, and Cr underscored the mounting influence of nutrient salts and heavy metals accumulation due to dams on the bacterioplankton taxa. This effect was particularly salient given the heightened urbanization and extensive agricultural land usage (Liu et al., 2023), which facilitated the inflow of domestic and agricultural wastewater into the river and its subsequent accumulation *via* the cascade dams (Zeng et al., 2022). This, in turn, accentuated water pollution and significantly molded the composition of bacterioplankton communities (Ma et al., 2019). The downstream cascade dams demonstrated heightened sensitivity of the bacterioplankton community structure to environmental variables. Water turbidity and TDS emerged as standout contributors. Notably, suspended solids and particulate matter surfaces acted as absorptive agents for organic pollutants, including polycyclic aromatic hydrocarbons. Elevated TDS concentrations consequently stimulated the proliferation of heterotrophic bacterioplankton, resulting in the emergence of distinct bacterioplankton communities proximal to the dams (Herfort et al., 2017). However, the turbidity associated with such conditions hindered light penetration, thereby posing challenges to the survival of phototrophic bacteria.

### 4.4. Relationship between environmental variables and bacterial functional groups

In addition to changes in community composition, it is essential to focus on variations in the function of bacterial communities. FAPROTAX has been found to be effective at predicting changes in bacterial ecological functional profiles across a range of environmental conditions (Louca et al., 2016; Gu et al., 2019). Our study revealed that the upstream region of cascade dams houses a significant number of functional bacterial groups that are engaged in nitrogen transformation. It is worth noting that only two key variables, ORP and WT, affected the construction of bacterioplankton taxa at upstream, while the functional community profiles showed noteworthy correlations with several environmental factors, such as EC, NTU, and the presence of heavy metals such as Pb, Cd, and Zn. It suggested that while the upstream bacterial community is structurally homogeneous, there is still rich in functional groups and tightly linked to the environment (Mai et al., 2022). In the midstream region, a marked augmentation was observed in the abundance of functional groups dedicated to the transformation of organic matter and carbon. This occurrence likely stems from intensified land utilization and the multi-tiered interception of water flow facilitated by the cascade dam system. This confluence accentuates the elevation of organic matter levels and specific heavy metal concentrations within the midstream (Shen et al., 2023). Significantly, these functional groups demonstrated significant correlations with numerous variables, such as water temperature, ORP, and specific metal content. This alignment with the outcomes presented in Section 3.3, where the midstream bacterial community structure was demonstrated to be significantly influenced by environmental factors, underscoring the coherence in how the structure and functional groups of midstream bacterioplankton in cascade dam systems react to heightened environmental pressures (Li et al., 2022). In the downstream, functional diversity was increased, especially photosynthetic bacteria，promoting material exchange and enhancing ecosystem stability (Allison and Martiny, 2008; Wang et al., 2020a). This validation corresponds with the conclusions drawn in Section 3.3, which emphasized that the bacterial community structure within downstream dams mirrors greater complexity and proximity to that of natural rivers. This further accentuates that the microbial community structure and functional diversity observed in the downstream reach of cascade dam systems closely approach the characteristics typical of natural river ecosystems.

### 4.5. Disparities in environmental responses between taxonomic diversity and functional groups and their interrelations

The SEM results indicated that at different regions in the cascade dams, environmental variables had different weights of influence on bacterioplankton functional groups and taxonomic diversity. Functional groups were more susceptible to water quality and heavy metals than taxa, as demonstrated in other cascade dams (Ofiţeru et al., 2010). We surmised that dams predominantly shape metabolic niches by modifying regional environments, resulting in distinctive distributions of functional groups (Louca et al., 2016). Notably, within the midstream of cascade dams, the negative correlations between environmental variables and community taxonomic and functional groups increased. These correlations suggested that city rivers regulated by dams have higher nutrient and heavy metal levels, which could lead to eutrophication and environmental stress (Liu et al., 2023). Consequently, adopting a proactive approach toward sewage effluent management within the midstream of cascade dams emerges as an imperative measure to uphold taxonomic and functional diversity.

Our results also indicated a significant association between community functional groups and taxonomic diversity, which has been corroborated by previous research (Louca et al., 2016). However, the presence of cascade dams has introduced considerable variability in the relationship between them at the regional scale. In the upstream, there was a notable negative correlation between β-diversity and functional groups involved in sulfur transformation. The simplified taxonomic structure of upstream bacterioplankton communities was contrasted by the abundance of functional groups related to sulfur cycling, indicating that upstream reservoirs indeed weakened both taxonomic and functional diversity within the river ecosystem. In the downstream, the increased diversity of taxonomic groups significantly contributed to the expansion of functional groups. This phenomenon could be attributed to the existence of many coexisting yet taxonomically distinct microorganisms that encoded the same energy-yielding metabolic functions, thereby fostering functional redundancy (Galand et al., 2018; Louca et al., 2018). This redundancy, in turn, amplified the overall stability of the river ecosystem.

## 5. Conclusion

Our findings demonstrated that cascade dams significantly alter the bacterioplankton community structure and function through changes in the river environment, with varying impacts in different regions. Future research should emphasize the regional-scale effects of cascade dams on ecosystems, particularly concerning water quality and heavy metal concentrations. For effectively managing cascade-regulated rivers, focus should be on enhancing upstream connectivity, increasing environmental heterogeneity, implementing pollution control measures in the midstream region, reducing pollutant retention and discharge, and strengthening monitoring efforts in the downstream region to prevent water quality degradation. The assessment of the bacterioplankton structure and functional diversity must be incorporated into monitoring and regulatory frameworks for informed decision-making.

## Data availability statement

The datasets presented in this study can be found in online repositories. The names of the repository/repositories and accession number(s) can be found at: https://www.ncbi.nlm.nih.gov/, PRJNA1005440.

## Author contributions

XJ: Conceptualization, Data curation, Investigation, Methodology, Software, Visualization, Writing – original draft. YL: Data curation, Investigation, Software, Writing – original draft. RZ: Data curation, Methodology, Writing – original draft. TS: Data curation, Investigation, Writing – original draft. JC: Data curation, Investigation, Writing – original draft. SA: Funding acquisition, Resources, Supervision, Writing – review & editing. JS: Writing – review & editing. XL: Funding acquisition, Project administration, Resources, Supervision, Writing – review & editing.

## Footnotes

^1^ https://www.thermofisher.com

^2^ www.majorbio.com
