# Supplementary material for: Cascade dams altered taxonomic and functional composition of bacterioplankton community at the regional scale
Source: Front Microbiol. 2023 Oct 25;14:1291464. doi: 10.3389/fmicb.2023.1291464 (PMC10634544; doi:10.3389/fmicb.2023.1291464)
Supplement: Supplementary file 1 [file Data_Sheet_1.pdf]

Supplementary Material

Cascade Dams Altered Taxonomic and Functional Composition of Bacterioplankton Community at the Regional Scale

Xufei Jiang[a], Yan Liu[a], Rixiu Zhou[a], Tianyi Sun[a], Jingdan Cao[a], Shuqing An[a], Jiachen Shen[b], Xin Leng[a*]

[a] School of Life Science and Institute of Wetland Ecology, Nanjing University, Nanjing, 210000, China.

[b] Key Laboratory for Information System of Mountainous Area and Protection of Ecological Environment of Guizhou Province, Guizhou Normal University, Guiyang, China.

***Correspondence:**
Xin Leng: lengx@nju.edu.cn

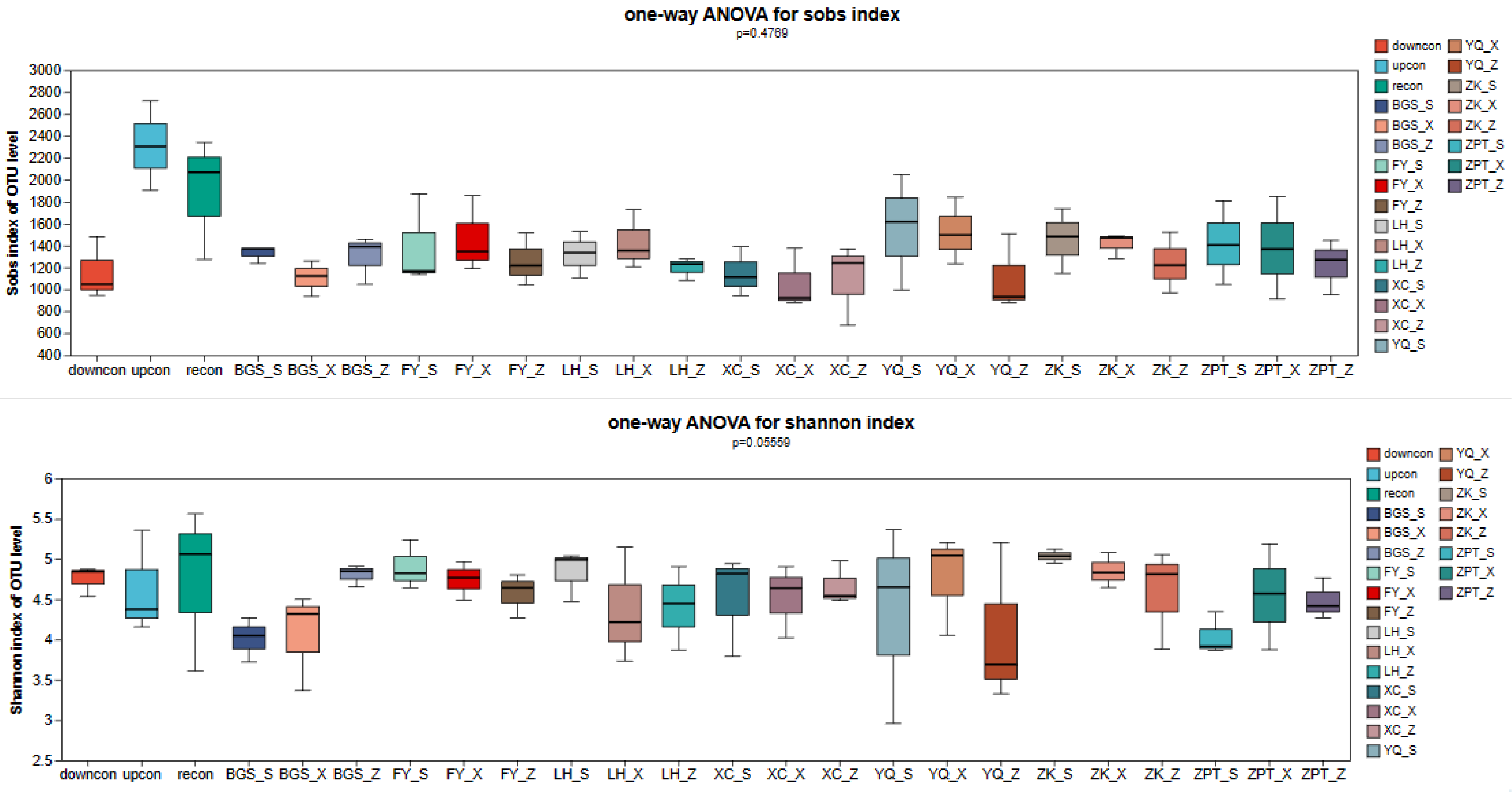

Figure S1. one-way ANOVA for upstream, midstream and downstream of each dam, for abundance and shannon diversity, respectively.

Table S1. Tukey HSD test for different locations of basin. Up means upstream of cascade dams, Mid represents midstream and Down means downstream, Approximate probabilities for Post Hoc Tests df = 60. Table shows p-values and red color indicates a significant correlation.

**WQ**	**PH**	**DO**	**WT**	**EC**	**ORP**	**TSS**	**NTU**	**RP**	**TP**	**TN**	**NH_4^+-N**	**COD**	**Hg**	**Pb**	**Cd**	**As**	**Zn**	**Cu**	**Fe**	**Cr**	**Al**
Up-Down	0.990	0.854	0.933	0.000	0.752	0.000	0.001	0.009	0.002	0.016	0.031	0.412	0.787	0.440	0.680	0.000	0.942	0.999	0.083	0.000	0.100
Up-Mid	0.972	0.962	0.978	0.000	0.257	0.000	0.063	0.712	0.119	0.000	0.149	0.916	0.595	0.950	0.035	0.000	0.755	0.719	0.187	0.001	0.161
Mid-Down	0.925	0.671	0.826	0.001	0.053	0.036	0.102	0.033	0.158	0.285	0.611	0.578	0.966	0.554	0.240	0.129	0.933	0.752	0.814	0.393	0.901

Table S2. Midstream and downstream keystone species screened in the co-occurrence network.

Phylum	Abundance	Betweenness Centrality	Degree	Class	Order	Family	Genus
					Midstream		
Actinobacteriota	3477	2324.42	118	Acidimicrobiia	Microtrichales	Ilumatobacteraceae	CL500-29_marine_group
Actinobacteriota	604	1892.88	115	Actinobacteria	Frankiales	Sporichthyaceae	hgcI_clade
Actinobacteriota	1087	2198.57	115	Acidimicrobiia	Microtrichales	Ilumatobacteraceae	CL500-29_marine_group
Actinobacteriota	3311	1667.17	113	Actinobacteria	Frankiales	Sporichthyaceae	hgcI_clade
Proteobacteria	1644	1103.06	104	Gammaproteobacteria	Burkholderiales	Comamonadaceae	Polaromonas
					Downstream		
Proteobacteria	531	1048.70	145	Gammaproteobacteria	Burkholderiales	Comamonadaceae	Rhodoferax
Proteobacteria	1132	882.71	140	Gammaproteobacteria	Burkholderiales	Methylophilaceae	norank_Methylophilaceae
Bacteroidota	484	1308.80	139	Kapabacteria	Kapabacteriales	norank_Kapabacteriales	norank_norank_Kapabacteriales
Actinobacteriota	473	994.00	138	Actinobacteria	Frankiales	Sporichthyaceae	hgcI_clade
Proteobacteria	454	757.66	134	Gammaproteobacteria	Gammaproteobacteria_Incertae_Sedis	unclassified_Gammaproteobacteria_Incertae_Sedis	Acidibacter
Bacteroidota	648	724.50	133	Bacteroidia	Flavobacteriales	Flavobacteriaceae	Flavobacterium
Proteobacteria	3120	1477.83	133	Alphaproteobacteria	Rhizobiales	Rhizobiaceae	Ochrobactrum
Actinobacteriota	115	1310.51	131	Actinobacteria	Micrococcales	Microbacteriaceae	Candidatus_Planktoluna
Actinobacteriota	248	1556.55	130	Actinobacteria	Frankiales	Sporichthyaceae	hgcI_clade
Proteobacteria	611	493.16	127	Alphaproteobacteria	Caulobacterales	Caulobacteraceae	Caulobacter
Proteobacteria	54	820.67	126	Gammaproteobacteria	Burkholderiales	Comamonadaceae	Rhodoferax

Actinobacteriota	3453	528.21	125	Actinobacteria	Frankiales	Sporichthyaceae	hgcI_clade
Actinobacteriota	175	548.46	123	Actinobacteria	Frankiales	Sporichthyaceae	hgcI_clade
Bacteroidota	186	607.19	120	Bacteroidia	Flavobacteriales	Flavobacteriaceae	Flavobacterium
Proteobacteria	1123	449.49	120	Gammaproteobacteria	Burkholderiales	Comamonadaceae	Polaromonas
Proteobacteria	415	777.92	119	Gammaproteobacteria	Burkholderiales	Burkholderiaceae	Polynucleobacter
Proteobacteria	930	446.77	119	Gammaproteobacteria	Burkholderiales	Methylophilaceae	unclassified_Methylophilaceae
Proteobacteria	236	604.15	119	Gammaproteobacteria	Burkholderiales	Oxalobacteraceae	norank_Oxalobacteraceae
Cyanobacteria	1060	526.13	117	Cyanobacteriia	Synechococcales	Cyanobiaceae	Cyanobium_PCC-6307
Proteobacteria	136	781.76	116	Gammaproteobacteria	Burkholderiales	T34	norank_T34
Actinobacteriota	731	902.40	115	Actinobacteria	PeM15	norank_PeM15	norank_norank_PeM15
Actinobacteriota	7374	834.48	114	Actinobacteria	Corynebacteriales	Nocardiaceae	Rhodococcus
Cyanobacteria	3011	505.94	114	Cyanobacteriia	Synechococcales	Cyanobiaceae	Cyanobium_PCC-6307
Actinobacteriota	3721	531.92	113	Actinobacteria	Frankiales	Sporichthyaceae	hgcI_clade
Proteobacteria	62	714.44	112	Gammaproteobacteria	Burkholderiales	Comamonadaceae	unclassified_Comamonadaceae
Actinobacteriota	5744	668.71	112	Actinobacteria	Frankiales	Sporichthyaceae	norank_Sporichthyaceae
Cyanobacteria	247	589.88	110	Cyanobacteriia	Synechococcales	Cyanobiaceae	Cyanobium_PCC-6307
Proteobacteria	419	804.93	109	Alphaproteobacteria	Rhodobacterales	Rhodobacteraceae	unclassified_Rhodobacteraceae
Cyanobacteria	1221	453.69	109	Cyanobacteriia	Synechococcales	Cyanobiaceae	Cyanobium_PCC-6307
Bacteroidota	471	677.74	109	Bacteroidia	Chitinophagales	Chitinophagaceae	Terrimonas
Proteobacteria	1536	1267.44	109	Gammaproteobacteria	Burkholderiales	Comamonadaceae	Limnohabitans
Proteobacteria	103	919.93	107	Gammaproteobacteria	Burkholderiales	Comamonadaceae	unclassified_Comamonadaceae

Verrucomicrobiota	265	454.20	107	Verrucomicrobiae	Methylacidiphilales	Methylacidiphilaceae	norank_Methylacidiphilaceae
Proteobacteria	517	645.48	107	Gammaproteobacteria	Burkholderiales	Methylophilaceae	Methylotenera
Proteobacteria	572	355.91	106	Gammaproteobacteria	Methylococcales	Methylomonadaceae	norank_Methylomonadaceae
Bacteroidota	176	637.68	106	Bacteroidia	Flavobacteriales	Flavobacteriaceae	Flavobacterium
Proteobacteria	285	585.00	106	Gammaproteobacteria	Burkholderiales	Comamonadaceae	Sphaerotilus
Bacteroidota	52	2271.62	105	Bacteroidia	Sphingobacteriales	env.OPS_17	norank_env.OPS_17
Bacteroidota	66	1844.25	105	Bacteroidia	Sphingobacteriales	Sphingobacteriaceae	Pedobacter
Proteobacteria	1739	1099.85	104	Gammaproteobacteria	Burkholderiales	Comamonadaceae	Delftia
Bacteroidota	544	414.85	104	Bacteroidia	Flavobacteriales	Crocinitomicaceae	Fluviicola
Bacteroidota	95	2012.86	103	Bacteroidia	Chitinophagales	Chitinophagaceae	Aurantisolimonas
Actinobacteriota	2066	540.90	102	Acidimicrobiia	Microtrichales	Ilumatobacteraceae	CL500-29_marine_group
Proteobacteria	485	1006.74	102	Gammaproteobacteria	Burkholderiales	Comamonadaceae	Limnohabitans
Verrucomicrobiota	67	1713.27	101	Verrucomicrobiae	Verrucomicrobiales	Verrucomicrobiaceae	norank_Verrucomicrobiaceae
Bacteroidota	99	2862.26	101	Bacteroidia	Flavobacteriales	Flavobacteriaceae	Flavobacterium
Actinobacteriota	92	482.05	100	Actinobacteria	Frankiales	Sporichthyaceae	hgcI_clade
Proteobacteria	922	739.51	100	Gammaproteobacteria	Burkholderiales	MWH-UniP1_aquatic_group	norank_MWH-UniP1_aquatic_group

Table S3. Explanatory rates of different environmental variables in the RDA analysis in upstream, midstream and downstream, with red indicating significant correlation.

Upstream					Midstream					Downstream				
**Environment variables**	**RDA1**	**RDA2**	**r2**	**p_values**	**Environment variables**	**RDA1**	**RDA2**	**r2**	**p_values**	**Environment variables**	**RDA1**	**RDA2**	**r2**	**p_values**
ORP	-0.02	1.00	0.64	0.00	WT	1.00	-0.10	0.62	0.00	PH	0.97	0.23	0.77	0.00
WT	-0.08	1.00	0.42	0.02	ORP	0.98	-0.17	0.50	0.00	NH3-N	-0.66	-0.75	0.74	0.00
Zn	0.44	-0.90	0.27	0.07	TN	-0.78	0.62	0.50	0.00	WT	0.95	0.31	0.84	0.00
Cd	0.08	-1.00	0.27	0.08	As	-0.94	0.34	0.54	0.00	TDS	-1.00	-0.07	0.56	0.00
Fe(ug/L)	0.16	-0.99	0.24	0.11	Cr	-0.98	0.20	0.51	0.00	Turbidity	-0.62	-0.78	0.58	0.00
RP	-0.98	-0.21	0.23	0.12	DO	-0.89	0.47	0.42	0.00	As	-0.98	-0.22	0.56	0.00
DO	0.58	-0.82	0.23	0.15	Cd	-0.93	0.38	0.32	0.01	RP	-1.00	-0.09	0.50	0.01
TN	-0.58	-0.81	0.21	0.18	COND	-0.63	0.78	0.26	0.03	COND	-0.76	-0.65	0.52	0.01
As	-0.20	-0.98	0.20	0.19	Turbidity	-0.77	0.64	0.24	0.03	ORP	1.00	0.09	0.52	0.01
Turbidity	0.67	0.74	0.17	0.23	TDS	-0.98	0.18	0.22	0.04	Cd	-0.80	-0.60	0.51	0.01
Al	-0.32	-0.95	0.16	0.28	Zn	-1.00	0.03	0.20	0.06	TN	-0.97	-0.25	0.50	0.01
Cr	-0.71	-0.71	0.14	0.32	TP	-0.29	0.96	0.18	0.09	Cr	-0.98	0.20	0.44	0.02
NH3-N	0.66	-0.76	0.10	0.37	Fe(ug/L)	-0.73	-0.68	0.14	0.12	Cu	-0.91	-0.41	0.42	0.02
Cu	-0.58	-0.81	0.12	0.39	Al	-0.83	-0.55	0.13	0.18	Zn	-0.82	-0.57	0.36	0.03
TDS	-0.61	-0.79	0.10	0.47	COD	0.99	0.14	0.11	0.23	Al	-0.68	0.74	0.32	0.06
COND	-0.20	-0.98	0.09	0.48	NH3-N	-0.92	0.39	0.09	0.33	Fe(ug/L)	-0.66	0.75	0.30	0.07
PH	0.62	0.79	0.01	0.92	Cu	0.73	0.68	0.07	0.41	DO	0.96	-0.27	0.21	0.20
					Pb	0.98	0.22	0.05	0.52					
					PH	-0.25	0.97	0.05	0.58					
					RP	-0.93	0.37	0.02	0.76					
					Hg	0.99	0.16	0.02	0.76					

Table S4. Heavy metal content of water at three periods in 21 dam sampling sites and 3 controls

Plot	Season	Dam	Hg (mg/L)	Pb (mg/L)	Cd (mg/L)	As (mg/L)	Zn (mg/L)	Cu (mg/L)	Fe (mg/L)	Cr (mg/L)	Al (mg/L)
B1	Jan	downstream control	0.000033	0.000023	0.000092	0.003802	0.005186	0.001671	0.002	0.004093	0.005298
B1_2	Aug	downstream control	0.001066	0.000065	0.000043	0.002914	0.000009	0.00253	0.018	0.002958	0.000094
B1_3	Nov	downstream control	0.000172	0.000012	0.000015	0.003352	0.000192	0.000973	0.045	0.004377	0.009982
B2	Jan	upstram control	0.000028	0.00002	0.000064	0.004185	0.001128	0.000967	0.014	0.00389	0.001746
B2_2	Aug	upstram control	0.002606	0.000008	0.000021	0.001199	0.001591	0.003073	0.005	0.001243	0.000329
B2_3	Nov	upstram control	0.000116	0.000011	0.000006	0.001767	0.000176	0.000266	0.028	0.002305	0.010897
B3	Jan	midstream control	0.000033	0.000007	0.000033	0.001633	0.0153	0.000919	0.016	0.001802	0.002678
B3_2	Aug	midstream control	0.000254	0.000018	0.000051	0.001994	0.003965	0.001502	0.008	0.002038	0.000039
B3_3	Nov	midstream control	0.000119	0.000044	0.000052	0.001778	0.02601	0.004148	0.042	0.004133	0.022215
BGS_up	Jan	BGS	0.000077	0.000014	0.000053	0.000717	0.003252	0.001463	0.024	0.000882	0.000744
BGS_up2	Aug	BGS	0.000112	0.000024	0.000008	0.000341	0.000014	0.000486	0.001	0.000385	0.000005
BGS_up3	Nov	BGS	0.000127	0.000103	0.000009	0.00088	0.000012	0.000818	0.042	0.001846	0.011405
BGS_down	Jan	BGS	0.000101	0.000009	0.000073	0.0015	0.005765	0.001085	0.02	0.001261	0.003777
BGS_down2	Aug	BGS	0.000189	0.000012	0.000016	0.000785	0.002604	0.000799	0.001	0.000829	0.001023
BGS_down3	Nov	BGS	0.000113	0.000105	0.000014	0.001731	0.000159	0.000459	0.036	0.001747	0.018819
BGS_mid	Jan	BGS	0.000939	0.000033	0.000066	0.00175	0.009369	0.00406	0.026	0.001151	0.027058
BGS_mid2	Aug	BGS	0.000394	0.000033	0.000013	0.000169	0.000648	0.001272	0.014	0.000213	0.00123
BGS_mid3	Nov	BGS	0.000129	0.000081	0.00001	0.00217	0.000031	0.000613	0.046	0.001816	0.039971
FY_up	Jan	FY	0.000114	0.000039	0.0001	0.003874	0.004723	0.002157	0.008	0.002678	0.001059
FY_up2	Aug	FY	0.000077	0.000024	0.000022	0.000875	0.000002	0.000483	0.009	0.000919	0.002028

FY_up3	Nov	FY	0.000234	0.000055	0.000035	0.003531	0.001443	0.001737	0.192	0.0042	0.181852
FY_down	Jan	FY	0.001018	0.000018	0.000073	0.00394	0.015585	0.006184	0.01	0.002399	0.001513
FY_down2	Aug	FY	0.000333	0.000046	0.000021	0.001745	0.003698	0.000851	0.022	0.001789	0.002145
FY_down3	Nov	FY	0.000175	0.000048	0.000048	0.003707	0.002768	0.003449	0.179	0.004297	0.191741
FY_mid	Jan	FY	0.001294	0.000007	0.000057	0.003374	0.00449	0.004844	0.008	0.00288	0.00531
FY_mid2	Aug	FY	0.000348	0.000013	0.000013	0.001659	0.000004	0.000374	0.011	0.001703	0.000002
FY_mid3	Nov	FY	0.000199	0.000097	0.000031	0.003521	0.001272	0.001778	0.212	0.004319	0.250446
LH_up	Jan	LH	0.000052	0.000004	0.000058	0.002031	0.010237	0.00172	0.017	0.00243	0.002805
LH_up2	Aug	LH	0.010346	0.00004	0.000016	0.000356	0.000004	0.011287	0.007	0.0004	0.000014
LH_up3	Nov	LH	0.000119	0.00002	0.000074	0.00197	0.000724	0.001036	0.121	0.002857	0.163776
LH_down	Jan	LH	0.000053	0.000007	0.000052	0.002054	0.010103	0.003959	0.012	0.002128	0.003984
LH_down2	Aug	LH	0.000665	0.000095	0.000022	0.000464	0.000454	0.001716	0.01	0.000508	0.000004
LH_down3	Nov	LH	0.000116	0.000048	0.000045	0.002041	0.000297	0.000817	0.092	0.002746	0.103118
LH_mid	Jan	LH	0.000043	0.000004	0.000046	0.002104	0.016756	0.001933	0.023	0.002111	0.003591
LH_mid2	Aug	LH	0.003592	0.000028	0.00002	0.000388	0.000001	0.004578	0.01	0.000432	0.00006
LH_mid3	Nov	LH	0.000111	0.000024	0.000051	0.002154	0.000834	0.001301	0.101	0.002918	0.11714
XC_up	Jan	XC	0.000053	0.000019	0.000072	0.003445	0.007961	0.002247	0.016	0.003487	0.005661
XC_up2	Aug	XC	0.002059	0.000055	0.000033	0.000944	0.000058	0.005204	0.019	0.000988	0.001496
XC_up3	Nov	XC	0.000125	0.000099	0.000073	0.002995	0.001792	0.001327	0.16	0.003851	0.174481
XC_down	Jan	XC	0.00004	0.000027	0.00009	0.003861	0.006282	0.002229	0.005	0.004194	0.005529
XC_down2	Aug	XC	0.000217	0.000044	0.000039	0.000807	0.000008	0.002079	0.015	0.000851	0.000061
XC_down3	Nov	XC	0.000206	0.000014	0.000053	0.002931	0.001802	0.001762	0.238	0.003711	0.284119
XC_mid	Jan	XC	0.000595	0.000027	0.00009	0.003767	0.005731	0.004139	0.001	0.004261	0.001936
XC_mid2	Aug	XC	0.0009	0.000045	0.000028	0.000894	0.000356	0.002495	0.013	0.000938	0.001415
XC_mid3	Nov	XC	0.000157	0.000046	0.000063	0.002815	0.003147	0.001016	0.145	0.003789	0.174134
YQ_up	Jan	YQ	0.000478	0.000009	0.00005	0.002087	0.003647	0.001175	0.018	0.002136	0.000122
YQ_up2	Aug	YQ	0.001112	0.000014	0.000016	0.001778	0.000003	0.000722	0.023	0.001822	0.004679
YQ_up3	Nov	YQ	0.000805	0.000067	0.000048	0.002699	0.0013	0.002474	0.126	0.004258	0.111441
YQ_down	Jan	YQ	0.001275	0.000005	0.000044	0.003536	0.004222	0.004197	0.001	0.003499	0.00812

YQ_down2	Aug	YQ	0.001403	0.000008	0.000031	0.001835	0.000001	0.000867	0.013	0.001879	0.000007
YQ_down3	Nov	YQ	0.000815	0.000009	0.000019	0.002908	0.002736	0.00228	0.268	0.004608	0.303605
YQ_mid	Jan	YQ	0.001311	0.00001	0.000068	0.003143	0.004181	0.004513	0.006	0.003014	0.002281
YQ_mid2	Aug	YQ	0.001416	0.000012	0.000018	0.001836	0.000012	0.000856	0.014	0.00188	0.000003
YQ_mid3	Nov	YQ	0.000654	0.000061	0.000022	0.002652	0.001957	0.000777	0.11	0.004423	0.108183
ZK_up	Jan	ZK	0.00006	0.000104	0.000151	0.004045	0.006062	0.003285	0.025	0.004387	0.016493
ZK_up2	Aug	ZK	0.000883	0.000035	0.000019	0.000672	0.003214	0.003586	0.011	0.000716	0.000054
ZK_up3	Nov	ZK	0.000138	0.000045	0.000053	0.002628	0.000776	0.000723	0.056	0.002919	0.02633
ZK_down	Jan	ZK	0.000035	0.000002	0.000077	0.003552	0.004442	0.00181	0.014	0.00359	0.003825
ZK_down2	Aug	ZK	0.000883	0.000121	0.000048	0.001721	0.001503	0.003459	0.043	0.001765	0.009808
ZK_down3	Nov	ZK	0.00013	0.000091	0.000042	0.002765	0.002305	0.001526	0.248	0.003639	0.324702
ZK_mid	Jan	ZK	0.000028	0.00001	0.000059	0.002478	0.002799	0.00174	0.009	0.002751	0.004812
ZK_mid2	Aug	ZK	0.00078	0.000021	0.000021	0.001067	0.000536	0.002904	0.013	0.001111	0.000002
ZK_mid3	Nov	ZK	0.00016	0.000022	0.000036	0.002798	0.000563	0.000947	0.087	0.003352	0.041304
ZPT_up	Jan	ZPT	0.000079	0.000005	0.000067	0.000726	0.004429	0.003237	0.03	0.00049	0.004672
ZPT_up2	Aug	ZPT	0.003158	0.000022	0.000022	0.000304	0.000002	0.005635	0.011	0.000348	0.000024
ZPT_up3	Nov	ZPT	0.00012	0.000092	0.000012	0.000341	0.000222	0.00069	0.01	0.001204	0.018868
ZPT_down	Jan	ZPT	0.000088	0.000083	0.00007	0.001394	0.007794	0.005085	0.004	0.00222	0.000991
ZPT_down2	Aug	ZPT	0.000557	0.000016	0.000013	0.000805	0.004749	0.002762	0.006	0.000849	0.000041
ZPT_down3	Nov	ZPT	0.000131	0.000033	0.000058	0.00091	0.001792	0.002762	0.033	0.002319	0.008729
ZPT_mid	Jan	ZPT	0.000786	0.000006	0.000055	0.001011	0.003766	0.004409	0.02	0.000414	0.005567
ZPT_mid2	Aug	ZPT	0.000171	0.000014	0.000013	0.000212	0.000006	0.001078	0.009	0.000256	0.000035
ZPT_mid3	Nov	ZPT	0.000115	0.000102	0.00001	0.001091	0.000197	0.002439	0.029	0.001229	0.012546
